# Investigation of the seroprevalence to equine coronavirus and SARS-CoV-2 in healthy adult horses recently imported to the United States

**DOI:** 10.1080/01652176.2023.2288876

**Published:** 2023-11-27

**Authors:** Nicola Pusterla, Kaila Lawton, Samantha Barnum

**Affiliations:** Department of Medicine and Epidemiology, School of Veterinary Medicine, University of California, Davis, CA, USA

**Keywords:** ECoV, SARS-CoV-2, serology, imported horses, prevalence factors

## Abstract

Adult horses are susceptible to equine coronavirus (ECoV) and severe acute respiratory syndrome coronavirus-2 (SARS-CoV-2), although, only ECoV has been linked to clinical disease. Little information is available regarding the seroprevalence against ECoV and SARS-CoV-2 in adult healthy horses. The goal of the present study was to determine the seroprevalence against two coronaviruses known to infect horses using convenience samples collected from horses recently imported from Europe to the United States from 2019 to 2023. A total of 385 banked serum samples were tested against ECoV and SARS-CoV-2 using previously validated ELISA assays. Prevalence factors including date of arrival in the United States, signalment and country of origin were available for the majority of the horses. A total of 9/385 (2.3%) and 4/385 (1.0%) horses tested seropositive for ECoV and SARS-CoV-2, respectively. The ECoV seropositive horses were all mares, ages 4 to 26 years (median 9 years) and originated from Germany, the Netherlands, Ireland, Belgium and Italy. These mares were predominantly imported during the summer and fall months. All SARS-CoV-2 seropositive horses were mares ages 5 to 10 years (median 7.5 years) imported from the Netherlands and the United Kingdom. The majority of the SARS-CoV-2 seropositive horses were imported during the colder months of the year. The study results support the presence of ECoV in Europe and report on the first SARS-CoV-2 seropositive healthy adult horses outside the United States. Commingling for movements by air and close contact to humans may predispose transmission with ECoV and SARS-CoV-2, respectively.

## 1. Introduction

In recent years, two beta coronaviruses with clinical and/or epidemiological relevance have emerged in adult equids. The clinically relevant equine coronavirus (ECoV), which was considered an enteric virus of foals for many decades (Davis et al. 2000; Guy et al. 2000), has recently been linked to fever, anorexia, lethargy with or without enteric signs (diarrhea, colic) in adult equids (Oue et al. 2011, 2013; Pusterla et al. 2013; Miszczak et al. 2014; Fielding et al. 2015; Berryhill et al. 2019; Bryan et al. 2019; Nemoto et al. 2019; Kambayashi et al. 2021; Hierweger et al. 2022). Infected equids generally develop blood work abnormalities consistent with a viral disease (leucopenie due to lymphopenia) and the diagnosis is supported through the PCR detection of ECoV in feces (Pusterla et al. 2013; Fielding et al. 2015; Berryhill et al. 2019). While ECoV infection in adult horses often remains self-limiting, rare but severe complications such as hyperammonemic encephalopathy and necrotizing enteritis have been reported (Giannitti et al. 2015). The epidemiology and risk factors associated with ECoV have remained poorly investigated. ECoV infections have been predominantly reported in adult racing, pleasure and performance horses during the colder months of the year. The seroprevalence to ECoV in population of healthy adult horses has been reported to range between 9.6 to 15.5% depending on the population tested, its geographic origin and time of the year (Kooijman et al. 2017; Schvartz et al. 2021; Kambayashi et al. 2023).

Equids are apparently susceptible to SARS-CoV-2 based on the affinity of the viral receptor ACE-2 to bind SARS-CoV-2 spike protein (Liu et al. 2021). Experimental and field studies have been unable to document clinical disease and viral recovery in horses (Bosco-Lauth et al. 2021; Pusterla et al. 2022; Lawton et al. 2022a, 2022b). However, antibodies specific to SARS-CoV-2 have been reported in healthy pleasure and racing horses with contact to COVID-19 individuals, supporting the hypothesis that horses can become infected through the spillover from infected humans (Pusterla et al. 2022; Lawton et al. 2022a). Seroprevalence to SARS-CoV-2 in population of healthy racing horses and hospitalized horses has been reported to range from 3.5 to 5.9%, with time of the year being a possible prevalence factor for seropositivity (Lawton et al. 2022a; 2022b).

Commingling and long-distance transportations of equids are apparent risk factors for the spread of contagious pathogens (Englund and Pringle 2004; Dominguez et al. 2016; Muscat et al. 2018). Further, such events are generally linked to greater biosecurity risks and interactions with humans, potentially increasing the risk of intra- and inter-species transmissions (Sack et al. 2020). Therefore, the aim of this study was to investigate the prevalence and selected risk factors associated with seropositivity against ECoV and SARS-CoV-2 in adult horses recently imported to the United States from Europe.

## 2. Materials and methods

### 2.1. Study population and sample collection

The study population was composed of 385 healthy adult horses recently imported from various European country to the United States from January of 2019 to May of 2023. Imported horses from Europe were housed in shipping barns for 2-3 weeks prior of being moved by air to the United States. Once in the United States, the horses spent approximately 48 h in USDA import quarantine upon arrival at Los Angeles International Airport, 12 h in transit to the contagious equine metritis (CEM) quarantine facility, and 12–18 h in CEM quarantine before sampling. As part of the routine quarantine protocol, horses entering the quarantine facility had a physical examination performed and blood collected for a complete blood cell count. Owner’s written consent was obtained to use minimal data from the study horses (signalment and country of origin) and serum samples for research and publication purposes. Serum samples were banked and kept at −80 °C. In order to determine possible differences in seroprevalence against SARS-CoV-2, pre-COVID-19 pandemic samples (2019) and samples during the COVID-19 pandemic (2020–2023) were enrolled in the present study. Whenever available, date of sample collection, signalment (age, sex, breed) and country of origin were recorded for risk factor assessment.

### 2.2. Sample collection and analysis

All serum samples were tested for IgG to ECoV using an ELISA based on a recombinant protein containing two immunodominant areas of the spike protein of ECoV, including the

area with the highest predicted antigenic sequence (Kooijman et al. 2016). Antibody detection against SARS-CoV-2 was performed using a previously validated ELISA assay (Lawton et al. 2022a), which targets the immunodominant receptor binding domain (RBD) of the SARS-CoV-2 spike protein. Positive and negative controls were included for both ECoV and SARS-CoV-2 assays and test specific cut-off values for positive samples were calculated using six times the standard deviation above the OD mean value of seronegative samples.

Descriptive analyses were performed to evaluate the seasonality, demographics, and country of origin. Categorical analyses were performed using Pearson’s chi-square test to determine the association between observations (season, age, breed, sex, country of origin) and serological status. All statistical analyses were performed using commercial software (Stata Statistical Software, Version 14, College Station, TX, USA) and statistical significance was set at *p* < 0.05.

## 3. Results

All 385 adult horses were considered healthy based on normal physical parameters on the day of blood sample collection. Available serum samples ranged from 41 to 103 per year (Table 1). The study population ranged in age from 2 to 26 years with a median of 8 years. Warmblood was the most common breed represented. The population was composed of 359 mares and 26 stallions. The country of origin included Germany (102), The Netherlands (94), Belgium (49), France (18), Ireland (14), Sweden (10), United Kingdom (9), Spain (8), Denmark (5), Italy (2) Hungary (1), the Czech Republic (1), Poland (1), Portugal (1) and Switzerland (1). For all 69 samples from 2019, the country of origin was not available. A greater number of horses were imported during the winter months compared to other seasons.

**Table 1.** Signalment (age, breed, sex), country of origin and season of import in 385 horses tested for antibodies against ECoV and SARS-CoV-2 from 2019 to 2023.

**Year** **(sample number)**	**2019** **(69)**	**2020** **(41)**	**2021** **(103)**	**2022** **(91)**	**2023** **(81)**	**Total** **(385)**
**Age (years)**						
Range (median)	6-19 (12)	3-12 (6)	1-13 (7)	2-26 (7)	3-15 (8)	2-26 (8)
**Sex**						
Mares	67	37	94	85	76	359
Stallions	2	4	9	6	5	26
**Breed**						
Warmblood	64	39	101	85	72	361
Spanish breeds	2	0	2	1	1	6
Pony breeds	1	1	0	4	3	9
Other breeds	2	1	0	1	5	9
**Country of origin**						
Germany	NA	12	40	31	19	102
The Netherlands	NA	14	35	20	25	94
Belgium	NA	5	9	16	19	49
France	NA	3	5	5	5	18
Ireland	NA	2	2	6	4	14
Sweden	NA	0	5	4	1	10
United Kingdom	NA	2	1	2	4	9
Spain	NA	1	5	1	1	8
Other European countries	NA	2	1	6	3	12
**Season**						
Winter (December-February)	20	9	40	30	37	136
Spring (March-May)	17	0	26	0	44	87
Summer (June-August)	18	0	37	8	0	63
Fall (September-November)	14	32	0	53	0	99
**Seropositivity**						
ECoV	1	0	0	7	1	9
SARS-CoV-2	0	0	1	0	3	4

NA = data not available.

A total of 9/385 (2.3%) and 4/385 (1.0%) horses tested seropositive for ECoV and SARS-CoV-2, respectively (Table 2). The nine ECoV seropositive horses were all mares, ages 4 to 26 years (median 9 years) and originated from Germany (3 horses), the Netherlands (2 horses), Ireland (1 horse), Belgium (1 horse) and Italy (1 horse). For one ECoV seropositive horse, the country of origin was unknown. These mares were predominantly imported during the summer (5 horses) and fall (3 horses) months. All four SARS-CoV-2 seropositive horses were mares ages 5 to 10 years (median 7.5 years) imported from the Netherlands (3 horses) and the United Kingdom (1 horse). The majority of the SARS-CoV-2 seropositive horses were imported during the colder months of the year.

**Table 2.** Signalment (age, sex breed), origin and season of import in horses testing seropositive to ECoV and SARS-CoV-2.

Seropositivity	Age (years)	Breed	Sex	Origin	Season
ECoV	10	Warmblood	Mare	NA	Summer 2019
	6	Warmblood	Mare	Germany	Summer 2022
	9	Pony	Mare	The Netherlands	Summer 2022
	9	Warmblood	Mare	Italy	Summer 2022
	6	Warmblood	Mare	Germany	Summer 2022
	9	Warmblood	Mare	The Netherlands	Fall 2022
	4	Warmblood	Mare	Ireland	Fall 2022
	26	Warmblood	Mare	Germany	Fall 2022
	9	Warmblood	Mare	Belgium	Spring 2023
SARS-CoV-2	8	Warmblood	Mare	The Netherlands	Summer 2021
	10	Warmblood	Mare	The Netherlands	Winter 2023
	5	Warmblood	Mare	The Netherlands	Winter 2023
	7	Warmblood	Mare	United Kingdom	Spring 2023

NA = data not available.

## 4. Discussion

Overall, the results showed that in a population of recently imported horses from various European countries, a small percentage of horses had detectable antibodies to ECoV or SARS-CoV-2. While both coronaviruses are closely related viruses, cross-reactivity has been previously ruled out and is further supported by the lack of any study horse testing seropositive for both viruses (Lawton et al. 2022a). ECoV infection in adult horses was first reported from an outbreak in draft horses at a racetrack in Tokachi, Hokkaido, Japan in the summer of 2009 (Oue et al. 2011). Since this first clinical report, ECoV infections in adult horses have been confirmed from the United States, Europe (France, United Kingdom, Switzerland, Ireland) and Japan (Oue et al. 2011, 2013; Pusterla et al. 2013; Miszczak et al. 2014; Fielding et al. 2015; Berryhill et al. 2019; Bryan et al. 2019; Nemoto et al. 2019; Kambayashi et al. 2021; Hierweger et al. 2022). While the focus of ECoV infection is predominantly on clinical disease, little work has been done regarding the epidemiology of this emerging enteric virus. The seroprevalence of the present study population was 2.3%, which is lower than previously reported seropositivity rates from the United States, Israel and Japan (Kooijman et al. 2017; Schvartz et al. 2021; Kambayashi et al. 2023). Of interest is the observation that the majority of the seropositive horses originated from countries (Germany, the Netherlands, Italy and Belgium) with no reported endemic occurrence of ECoV infections in adult horses. One ECoV seropositive horse came from Ireland, a country with recently reported clinical cases of ECoV in foals and adult horses (Nemoto et al. 2019). Assuming that ECoV is endemic across all the imported countries, variations in susceptibility and infection pressure may have accounted for differences in the number of ECoV seropositive horses per country. Geographic region, age, breed, sex and use are risk factors previously reported to be associated with ECoV seropositivity (Kooijman et al. 2017; Schvartz et al. 2021; Kambayashi et al. 2023). Because the present study population was predominantly composed of young Warmblood mares, there is not enough contrast to draw any conclusions on age, breed and sex as possible risk factors linked to ECoV seropositivity. Unfortunately, the intended use of the recently imported horses was unknown to the investigators, which is another limitation of the present study. Because the study horses were only tested once, shortly after arrival to the United States, and previous medical records were unavailable, it was not possible to determine when seropositivity was acquired and if ECoV infection was associated with clinical or subclinical disease. Experimental infections with ECoV have shown that seroconversion can be expected as early as 14 days post exposure, however, the duration of detectable antibodies to ECoV has not yet been reported (Nemoto et al. 2014; Schaefer et al. 2018). In experimental infections using the closely related bovine coronavirus (BCoV), adult cows had detectable antibodies against BCoV up to 22 months (Tråvén et al. 2001). Time of infection and duration of detectable antibodies to ECoV are relevant factors in epidemiological studies using a single time point measurement. While ECoV infections are diagnosed year-round, greater frequency of clinical disease has been reported during the colder months of the year, probably driven by husbandry practices and environmental factors predisposing spread (Pusterla et al. 2013). Because the majority of the ECoV seropositive study horses were imported during the warmer months of the year (summer and fall), it is possible that clinical or subclinical infection occurred during the colder seasons preceding the movement of these horses.

The seroprevalence to SARS-CoV-2 was even lower with 1% compared to the seroprevalence against ECoV. Pervious seroepidemiological studies have shown no to very low seropositivity in convenience samples from healthy horses from China and the United States (Deng et al. 2020; Pusterla et al. 2022; Lawton et al. 2022a, 2022b). The transmission route for SARS-CoV-2 in equids has remained speculative but likely occurs *via* the spillover from COVID-19 patients (Pusterla et al. 2022). Horses that are prepared for long distance shipping generally experience numerous interactions with handlers, care takers and health care providers. This scenario, while not quantitate in the present study, is a predisposing factor for the spread of SARS-CoV-2 from individuals to horses as previously shown for racing horses and also for hospitalized patients (Lawton et al. 2022a; 2022b). The COVID-19 pandemic was declared in 2019 and expired in May of 2023 (https://www.hhs.gov/about/news/2023/05/09/fact-sheet-end-of-the-covid-19-public-health-emergency.html). Therefore, SARS-CoV-2 seropositive horses were not expected until the various country specific regulatory restrictions were lifted. Further, horse movements by air were dramatically reduced with the 2020 start of the global COVID-19 pandemic and only regained momentum in 2021 (Felici et al. 2023). This may explain why SARS-CoV-2 seropositive imported horses were first detected in 2021 and most of them observed in 2023. One explanation for the increased observation of SARS-CoV-2 seropositive horses in 2023 compared to the previous years is the circulation of new variants (Omicron variant), which are characterized by a greater rate of virus transmission (Kannan et al. 2021). While the number of SARS-CoV-2 seropositive horses is too small to calculate any relevant risk factors, the four seropositive horses are the first evidence of equine infections from Europe. Similar to any other susceptible animal species, SARS-CoV-2 natural infections and seropositivity are expected to be seasonal with greater infection rate during the colder months of the year. While a measurable antibody response post-SARS-CoV-2 infection is expected to rise rapidly, it is unknown how long specific antibodies persist in naturally infected animals. It is also possible, as shown for human patients, that a more rapid decrease in antibody levels occurs in patients with asymptomatic COVID-19 compared to patients with clinical disease (Long et al. 2020). Measurable antibodies to SARS-CoV-2 have been shown to persist up to 10-12 months in some dogs and cats from COVID-19 positive households (Decaro et al. 2022; Villanueva-Saz et al. 2023). Therefore, the authors speculate that the four SARS-CoV-2 seropositive horses had likely been infected close to or during the import season.

One of the limitations of the study is the nature of the study population considering that imported horses are generally young healthy performance horses and do not reflect the general population of horses from the various European import countries. The data may be, therefore, skewed towards a group of elite horses with greater risk of coronavirus infection due to regular human interactions and commingling practices. Another limitation is the lack of medical history for the study horses precluding any association between seropositive status and disease form (i.e. clinical versus subclinical). Last but not least, the lack of longitudinal data, especially in the case of subclinical infections, made it impossible to draw any conclusions regarding time of infection and risk of recent transportation.

In conclusion, the study results support the presence of ECoV in Europe and report on the first SARS-CoV-2 seropositive healthy adult horses outside the United States. While little comparative data is available for various horse populations, it appears that horses intended for export have husbandry and management practices that predispose infections with coronaviruses.
